# 异基因造血干细胞移植患者血流感染病原菌谱及耐药性——单中心回顾性分析

**DOI:** 10.3760/cma.j.issn.0253-2727.2021.10.003

**Published:** 2021-10

**Authors:** 丽敏 丁, 晓露 宋, 晓刚 王, 也 彭, 一瑞 陈, 莱 金, 建平 蓝

**Affiliations:** 1 蚌埠医学院临床医学系研究生院，蚌埠 233030 Graduate School of Clinical Medicine, Bengbu Medical College, Bengbu 233030, China; 2 浙江省人民医院血液病科和造血干细胞移植中心，杭州医学院附属人民医院 310014 Department of Hematology and Hematopoietic Stem Cell Transplant Center, Zhejiang Provincial People's Hospital, People's Hospital of Hangzhou Medical College, Hangzhou 310014, China; 3 浙江省肿瘤分子诊断与个体化治疗研究重点实验室，杭州 310014 Key Laboratory of Tumor Molecular Diagnosis and Individualized Medicine of Zhejiang Province, Hangzhou 310014, China

**Keywords:** 异基因造血干细胞移植, 血流感染, 病原菌, 耐药性, 生存期, Allogeneic hematopoietic stem cell transplantation, Bloodstream infection, Pathogenic Microorganism, Resistance, Survival

## Abstract

**目的:**

了解浙江省人民医院异基因造血干细胞移植（allo-HSCT）患者血流感染临床特点、病原菌谱、耐药性及危险因素。

**方法:**

回顾性分析2014年10月至2019年9月浙江省人民医院血液科210例allo-HSCT患者中发生血流感染的病原学分布、耐药性、感染危险因素及转归。

**结果:**

210例allo-HSCT患者中49例发生血流感染，共分离出病原菌59株，以革兰阴性菌为主（67.8％），其中以大肠埃希菌最为常见（23.7％），碳青霉烯类耐药革兰阴性菌（CRO）占42.5％；革兰阳性菌占23.7％，未发现万古霉素及利奈唑胺耐药的葡萄球菌株；真菌占8.5％。单因素分析提示血流感染影响因素有性别、移植前疾病状态、预处理方案；多因素分析显示血流感染主要与预处理方案强度有关（*OR*＝3.043，95％*CI* 1.236～7.492，*P*＝0.015）。49例血流感染患者中粒细胞缺乏（粒缺）血流感染占77.6％，非粒缺占22.4％；移植前活动性感染患者发生血流感染占81.0％，移植前无活动性感染患者占16.9％。生存分析结果显示，血流感染患者总生存（OS）时间短于未发生血流感染患者，CRO感染患者比无CRO感染患者OS时间短，粒缺时间>14 d患者OS时间较粒缺时间≤14 d患者短，移植前是否合并活动性感染及感染时是否处于粒缺状态与OS时间无明显相关性。

**结论:**

本研究提示积极预防耐药菌血流感染、缩短粒缺时间、移植前控制感染、适合的预处理方案，能降低移植死亡率、改善预后。

## Abstract (en)

**Objective:**

To elucidate the clinical characteristics of bloodstream infection in patients with allogeneic hematopoietic stem cell transplantation (allo-HSCT) in our hospital and improves the survival of transplant patients with bloodstream infection.

**Methods:**

Two hundred and ten patients with allo-HSCT from the Department of Hematology were retrospectively analyzed between October 2014 and September 2019. Pathogen distribution, drug resistance, risk factors, and outcomes were investigated in 49allo-HSCT patients with bloodstream infections.

**Results:**

Forty-nine of 210 patients with allo-HSCT had bloodstream infection, and 59 pathogenic microorganisms were identified, mainly Gram-negative bacteria (67.8％), of which E. coli had the highest incidence (23.7％), CRO accounted for 42.5％, and Gram-positive bacteria accounted for 23.7％ (without vancomycin or linezolid-resistant strain). Additionally, fungi accounted for 8.5％. Univariate analysis suggested that the risk factors of bloodstream infection were gender, pretransplant disease status, and conditioning regimen. In contrast, multivariate analysis showed that bloodstream infection was mainly related to conditioning regimens. Further grouping results showed that 77.6％ of patients with neutropenia had bloodstream infections, and 22.4％ of patients with non-neutropenia had bloodstream infections; 81.0％ of patients with active infections before transplantation had bloodstream infections, while bloodstream infection occurred in 16.9％ of patients without active infection. Survival analysis showed that long-term survival of patients with bloodstream infection is shorter than that of patients without bloodstream infection and long-term survival of patients with CRO infection is shorter than that of patients without CRO infection. The survival of patients with neutropenia longer than 14 d is shorter than that of patients with neutropenia shorter than 14 d. Furthermore, there is no correlation between whether there is an active infection before transplantation and whether they are in a neutropenic state at the time of infection and survival.

**Conclusion:**

Our results suggest that effective prevention of bloodstream infections from drug-resistant bacteria, particularly CRO, shortening the duration of neutropenia, eradication of potential infections before transplantation, and patient-adaptive conditioning could reduce transplant-related mortality and improve prognosis.

异基因造血干细胞移植（allo-HSCT）是血液系统恶性肿瘤和骨髓衰竭性疾病的重要治疗措施[1]。移植前化疗过程中长期反复住院以及预防性使用广谱抗生素易导致病原菌出现耐药[2]–[3]。而allo-HSCT患者移植过程中免疫力下降，容易引发血流感染，且一旦发生血流感染（尤其是耐药菌感染）预后极差[4]。为了解我院allo-HSCT患者血流感染临床特点，我们对本院210例allo-HSCT患者中49例发生血流感染患者的病原菌谱、耐药性、危险因素进行回顾性分析，现报道如下。

## 病例与方法

1. 病例资料：回顾性分析2014年10月至2019年9月浙江省人民医院血液科接受allo-HSCT的210例患者，女89例，男121例。主要疾病类型：急性髓系白血病（AML）97例、急性淋巴细胞白血病（ALL）54例、骨髓增生异常综合征（MDS）13例、非霍奇金淋巴瘤（NHL）15例、慢性髓性白血病（CML）15例。血培养病原菌剔除同一患者的重复分离菌。

2. 诊断标准与定义：（1）血流感染诊断标准：①在移植中或移植后体温>38.0 °C或<36.0 °C，可伴寒战，使用抗菌药物之前或高热寒战时完成需氧菌、厌氧菌和真菌培养；②血培养分离出病原微生物，若为常见皮肤菌，如类白喉棒状杆菌、丙酸杆菌等，需在不同时间采血，2次或多次培养阳性；③血培养中检出病原菌的抗原物质，与症状、体征相符[5]。（2）移植前活动性感染诊断标准：①预处理开始前体温>37.5°C伴炎症指标增高，并排除肿瘤性发热；②移植前1个月内有明确病原菌感染伴超敏C反应蛋白或降钙素原升高；③移植时影像学证实的感染灶（肺部、肝脏、脾脏）。（3）粒细胞缺乏（粒缺）：外周血中性粒细胞绝对计数（ANC）<0.5×10^9^/L或预计48 h后ANC<0.5×10^9^/L[6]。（4）总生存（OS）期：患者从预处理开始到死亡或随访终点的时间。（5）碳青霉烯类药物耐药的肠杆菌科细菌（CRE）、碳青霉烯类耐药革兰阴性菌（CRO）定义参照文献[7]标准。

3. 随访：随访截止时间为2019年9月，中位随访6（0～72）个月，共有12例患者失访。

4. 统计学处理：应用SPSS 17.0进行统计学分析。病原谱分布的组间比较采用Fisher精确概率法，采用Logistic回归对allo-HSCT后血流感染的影响因素进行单因素与多因素分析。采用Kaplan-Meier法绘制生存曲线，组间比较采用Log-rank检验。*P*<0.05为差异有统计学意义。

## 结果

一、血流感染影响因素

210例allo-HSCT患者中共有49例发生血流感染，女14例，男35例，中位年龄31（14～58）岁。血流感染的影响因素见表1，单因素分析结果显示性别、移植前疾病状态、预处理方案是血流感染的影响因素（*P*值均<0.05）；年龄、疾病类型、移植类型、粒缺持续时间等对血流感染的影响差异无统计意义。将单因素分析中*P*<0.1的因素纳入多因素分析，结果显示高强度预处理方案是血流感染的独立危险因素（*OR*＝3.043，95％ *CI* 1.236～7.492，*P*＝0.015）（表1）。

**表1:** 210例异基因造血干细胞移植患者血流感染影响因素分析

因素	例数	血流感染［例（％）］	单因素分析			多因素分析		
			*OR*	95％*CI*	*P*值	*OR*	95％*CI*	*P*值
性别			0.459	0.229～0.917	0.027	0.500	0.241～1.036	0.062
男性	121	35（28.9）						
女性	89	14（15.7）						
年龄			0.915	0.482～1.737	0.785	0.878	0.436～1.765	0.714
≤30岁	95	23（24.1）						
>30岁	115	26（22.6）						
移植前疾病状态			2.859	1.477～5.531	0.002	1.322	0.530～3.297	0.549
CR	142	24（16.2）						
非CR	68	25（38.8）						
疾病类型			1.110	0.918～1.344	0.282	1.023	0.832～1.257	0.832
AML	97	20（20.6）						
ALL	54	12（22.2）						
CML	15	5（33.3）						
MDS	13	3（23.1）						
NHL	15	4（26.7）						
其他	16	5（31.3）						
移植类型			0.896	0.500～1.608	0.712	0.937	0.496～1.768	0.841
亲缘全相合移植	56	14（25.0）						
亲缘半相合移植	145	34（23.4）						
非亲缘全相合移植	9	1（11.1）						
粒缺持续时间			1.144	0.574～2.281	0.703	1.219	0.575～2.584	0.605
<14d	69	15（21.7）						
>14d	141	34（24.1）						
移植前化疗次数			0.876	0.427～1.798	0.718	0.697	0.310～1.564	0.381
≤5次	150	36（24.0）						
>5次	60	13（21.7）						
预处理方案			3.972	2.032～7.765	<0.001	3.043	1.236～7.492	0.015
标准预处理	145	22（15.2）						
高强度预处理	65	27（41.5）						
发病至移植时间			1.809	0.940～3.481	0.076	1.492	0.709～3.140	0.292
≤10个月	138	27（19.6）						
>10个月	72	22（30.6）						

注：CR：完全缓解；AML：急性髓系白血病；ALL：急性淋巴细胞白血病；CML：慢性髓性白血病；MDS：骨髓增生异常综合征；NHL：非霍奇金淋巴瘤；粒缺：粒细胞缺乏；标准预处理：BUCY（白消安3.2 mg·kg^−1^·d^−1^，−7～−4 d；环磷酰胺60 mg·kg^−1^·d^−1^，−3～−2 d）、Ara-C+BUCY（阿糖胞苷4 g·m^−2^·d^−1^，−9～−8 d；白消安3.2 mg·kg^−1^·d^−1^，−7～−5 d；环磷酰胺1.8 g·m^−2^·d^−1^，−4～−3 d）；高强度预处理：Flu+CTX（氟达拉滨30 mg·m^−2^·d^−1^，−7～−2 d；环磷酰胺60 mg·kg^−1^·d^−1^，−2～−1 d；抗胸腺细胞球蛋白2.0 mg·kg^−1^·d^−1^，−4～−1 d）、FLAG+BUCY（氟达拉滨25 mg·m^−2^·d^−1^，−13～−9 d；阿糖胞苷2 g·m^−2^·d^−1^，−13～−9 d；G-CSF 300 µg/d，−13～−9 d；白消安3.2 mg·kg^−1^·d^−1^，−7～−5 d；环磷酰胺1.8 g·m^−2^·d^−1^，−4～−3 d；司莫司汀250 mg/m^2^，−2 d）、CLAG+BUCY（克拉屈滨5 mg·m^−2^·d^−1^，−13～−9 d；阿糖胞苷2 g·m^−2^·d^−1^，−13～−9 d；G-CSF 300 µg/d，−13～−9 d；白消安3.2 mg·kg^−1^·d^−1^，−7～−5 d；环磷酰胺1.8 g·m^−2^·d^−1^，−4～−3 d；司莫司汀250 mg/m^2^，−2 d）

二、病原菌分布

59株分离菌中细菌54株、真菌5株。其中，革兰阴性菌40株（67.8％），前3位分别为大肠埃希菌（23.7％）、肺炎克雷伯菌（20.3％）、铜绿假单胞菌（13.6％）；革兰阳性菌14株（23.7％），主要为葡萄球菌，未培养出金黄色葡萄球菌；真菌5株（8.5％），主要为热带念珠菌（3株，5.1％）（表2）。

**表2:** 59株异基因造血干细胞移植患者血流感染病原菌分布

病原菌类型	株数	构成比（％）
革兰阴性菌	40	67.8
大肠埃希菌	14	23.7
肺炎克雷伯菌	12	20.3
铜绿假单胞菌	8	13.6
奇异变形杆菌	2	3.4
其他阴性杆菌	4	6.8
革兰阳性菌	14	23.7
人型葡萄球菌亚种	4	6.8
溶血葡萄球菌	4	6.8
表皮葡萄球菌	1	1.7
松鼠葡萄球菌缓慢亚种	1	1.7
头状葡萄球菌亚种	1	1.7
屎肠球菌	1	1.7
缓症链球菌	1	1.7
蜡样芽孢杆菌	1	1.7
真菌	5	8.5
热带念珠菌	3	5.1
星状丝孢酵母菌	1	1.7
茄病镰刀菌	1	1.7

移植前21例患者伴活动性感染，17例（81.0％）发生血流感染，检出革兰阴性菌16株，革兰阳性菌3株，真菌3株；移植前无活动性感染患者189例，32例（16.9％）发生血流感染，检出革兰阴性菌24株，革兰阳性菌11株，真菌2株。两组病原菌谱分布差异无统计学意义（*P*＝0.312）。

49例血流感染患者中，粒缺期血流感染占77.6％（38例）；非粒缺期血流感染占22.4 ％（11例）。粒缺期血流感染病原菌革兰阴性菌34株，革兰阳性菌9株；非粒缺期血流感染病原菌革兰阴性菌6株，革兰阳性菌5株；大肠埃希菌和真菌仅见粒缺期血流感染。

三、药敏试验

1. 革兰阴性菌：14株大肠埃希菌中产超广谱β-内酰胺酶（ESBL）菌株共13株（92.9％），无CRE菌株；12株肺炎克雷伯菌中11株产ESBL，CRE 9株；CRE菌检出率为18.4％；检出CRO 17株，CRO菌株检出率为34.7％。其中，8株铜绿假单胞菌全为CRO菌株。CRO菌株对常见抗菌药物耐药性较高，对阿米卡星、妥布霉素敏感率较高，分别为82.4％、70.6％，其余均低于10.0％（表3）。大肠埃希菌对阿米卡星、厄他培南、亚胺培南、替加环素、哌拉西林/他唑巴坦耐药率较低，对头孢菌素类及喹诺酮类耐药率均较高。肺炎克雷伯菌对阿米卡星耐药率最低，为25％。铜绿假单胞菌对阿米卡星、妥布霉素耐药率最低，均未检测出耐药菌株（表4）。

**表3:** 17株碳青霉烯类耐药革兰阴性菌对抗菌药物的敏感率及耐药率（％）

抗菌药物	敏感率	耐药率
阿米卡星	82.4	17.6
头孢他啶	5.9	94.1
环丙沙星	5.9	82.4
头孢哌酮/舒巴坦	5.9	88.2
头孢吡肟	5.9	88.2
庆大霉素	52.9	41.2
亚胺培南	0	100.0
妥布霉素	70.6	17.6
哌拉西林/他唑巴坦	5.9	94.1

**表4:** 主要革兰阴性菌对抗菌药物的耐药率（％）

抗菌药物	大肠埃希菌（14株）	肺炎克雷伯菌（12株）	铜绿假单胞菌（8株）
阿米卡星	7.1	25.0	0
阿莫西林	27.2	80.0	–
氨曲南	85.7	75.0	–
头孢他啶	78.6	90.9	87.5
环丙沙星	85.7	66.7	87.5
头孢曲松	92.9	91.7	–
头孢哌酮/舒巴坦	33.3	81.2	87.5
头孢唑林	92.9	100.0	–
厄他培南	0	72.7	–
头孢吡肟	61.5	75.0	87.5
头孢西丁	54.5	70.0	–
庆大霉素	50.0	58.3	12.5
亚胺培南	0	75.0	100.0
左氧氟沙星	71.4	50.0	87.5
美洛培南	–	87.5	100.0
替加环素	0	45.4	–
复方磺胺甲恶唑	84.6	75.0	–
妥布霉素	21.4	33.3	0
哌拉西林/他唑巴坦	7.1	83.3	87.5

注：–：未做

2. 革兰阳性菌：所有革兰阳性葡萄球菌对利奈唑胺、万古霉素、替加环素的耐药率为0；对红霉素、青霉素G耐药率为100.0％（表5）。11株葡萄球菌中10株（90.9％）为耐甲氧西林葡萄球菌，屎肠球菌（1株）对万古霉素、替加环素、利奈唑胺均敏感。

**表5:** 主要革兰阳性葡萄球菌对抗菌药物的耐药率（％）

抗菌药物	人型葡萄球菌（4株）	溶血葡萄球菌（4株）	其他葡萄球菌（3株）
环丙沙星	100.0	100.0	66.7
克林霉素	100.0	0	100.0
红霉素	100.0	100.0	100.0
庆大霉素	0	50.0	0
利奈唑胺	0	0	0
左旋氧氟沙星	100.0	100.0	66.7
莫西沙星	100.0	75.0	66.7
呋喃妥因	0	100.0	0
苯唑西林	75.0	100.0	100.0
青霉素G	100.0	100.0	100.0
奎奴普丁/达福普汀	0	0	0
利福平	0	25.0	0
复方磺胺甲恶唑	100.0	50.0	66.7
四环素	100.0	75.0	33.3
万古霉素	0	0	0
替加环素	0	0	0

3. 真菌：共分离出真菌5株，其中热带念珠菌3株，星状丝孢酵母菌1株，茄病镰刀菌1株。3株热带念珠菌中2株对两性霉素B、5-氟胞嘧啶敏感，对氟康唑、伊曲康唑、伏立康唑耐药；1株对以上抗菌药均敏感。1株星状丝孢酵母菌对两性霉素B敏感，对唑类耐药。茄病镰刀菌未行药敏检测。

4. 粒缺期亚组分析：粒缺期发生血流感染患者中肺炎克雷伯菌10株，对碳青霉烯类耐药率为70.0％，阿米卡星耐药率为20.0％；非粒缺期发生血流感染患者肺炎克雷伯菌对碳青霉烯类耐药率为100.0％，对阿米卡星耐药率为50.0％。铜绿假单胞菌在粒缺期及非粒缺期发生时对阿米卡星均敏感，对碳青霉烯类均耐药。

四、转归

49例血流感染患者中18例因感染未有效控制死亡，18例患者中共培养出22株病原菌，其中3例患者合并多种病原菌感染。铜绿假单胞菌6例、肺炎克雷伯菌3例、人型葡萄球菌亚种1例、热带念珠菌2例、大肠埃希菌、茄病镰刀菌、多噬伯克霍尔德菌各1例；1例患者铜绿假单胞菌合并奇异变形杆菌，1例肺炎克雷伯菌合并人型葡萄球菌亚种，1例合并铜绿假单胞菌、肺炎克雷伯菌及奇异变形杆菌。另31例患者感染控制，后因其他病因死亡14例，目前存活17例。

生存分析显示血流感染、CRO血流感染患者OS率分别低于无血流感染、非CRO血流感染患者［（26.2±7.4）％对（55.7±4.6）％，*χ*^2^＝13.844，*P*<0.001；（7.5±7.1）％对（38.8±9.7）％，*χ*^2^＝8.804，*P*＝0.003］。移植前活动性感染患者与移植前无活动性感染患者OS差异无统计学意义［（28.2 ±13.1）％对（23.9±8.7）％，*χ*^2^＝0.111，*P*＝0.739］。粒缺期及非粒缺期发生血流感染患者OS差异无统计学意义［（33.2±9.1）％对（9.1±8.7）％，*χ*^2^＝0.516，*P*＝0.473］。然而，粒缺持续时间≤14 d患者OS率高于粒缺持续时间>14 d患者，差异有统计学意义［（46.7 ± 12.9）％对（12.9 ± 7.6）％，*χ*^2^＝4.998，*P*＝0.025］（图1）。

**图1:**
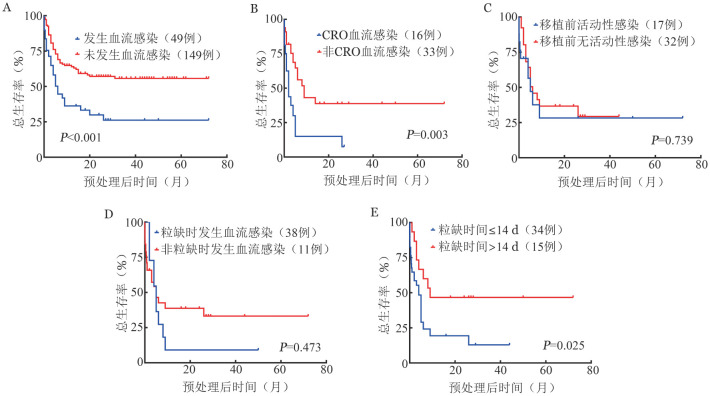
发生血流感染与否及不同疾病状态下发生血流感染异基因造血干细胞移植患者生存曲线比较 A：血流感染；B：碳青霉烯类耐药革兰阴性菌（CRO）血流感染；C：移植前活动性感染；D：粒细胞缺乏（粒缺）；E：粒缺持续时间>14 d

## 讨论

allo-HSCT患者预处理后多伴有粒缺，感染发生率显著升高[8]。不同地区、不同病房血流感染病原菌种类及耐药性存在差异[9]。血液系统疾病患者化疗相关血流感染中，常见的病原菌多为革兰阴性菌，对阿米卡星、头孢哌酮/舒巴坦、亚胺培南、哌拉西林/他唑巴坦、妥布霉素的总体耐药率较低，对头孢类抗菌药物呈现较高的耐药率[10]–[12]。Omrani等[13]研究提示，移植患者发生的血流感染中病原菌耐药率高于非移植患者，其中大部分对广谱β-内酰胺类、碳青霉烯类等抗菌药物具有耐药性。因此对allo-HSCT患者血流感染中病原菌的调查及耐药性分析具有重要意义。

本研究单因素分析结果提示性别、移植前疾病状态、预处理方案是allo-HSCT患者血流感染发生的影响因素，与文献[14]报道相似。进一步多因素分析结果显示allo-HSCT患者血流感染主要与预处理方案有关。高强度的预处理增加血流感染发生率，可能是高强度的预处理具有更强烈的骨髓抑制作用，延长粒缺时间导致。

相关研究[15]–[21]显示，allo-HSCT患者血流感染的致病菌主要为革兰阴性菌，allo-HSCT患者中CRE菌株占所有细菌的15.8％～23.7％，本研究结果与之相似。但本研究CRO检出率较高。除大肠埃希菌无CRO菌株外，肺炎克雷伯菌的CRE及铜绿假单胞菌的CRO菌株检出率分别为75.0％和100.0％，均较高。原因可能是本研究中有超过30％（68/210）的患者移植前未达CR，且142例CR患者中有24例移植前感染未完全控制，此两类患者均有长期广谱抗生素暴露史，部分患者此前已有CRO病原菌血流感染或定植史。

本研究中革兰阳性菌占23.7％，革兰阳性菌中以葡萄球菌为主；90.9％为耐甲氧西林葡萄球菌，对一般药物的耐药性较高，耐药率高于文献[22]报道，可能仍与移植前长期抗生素暴露相关。本组患者中真菌感染发生率较低（8.5％），真菌对两性霉素B耐药率为0，对唑类耐药率较高（75％）。真菌对两性霉素B较高的敏感性可能与前期无两性霉素B暴露相关，相较之下，使用较多的唑类抗真菌药的耐药率更高。

不同疾病状态的allo-HSCT患者发生血流感染的转归不同。Mikulska等[23]研究显示allo-HSCT不同细菌血流感染7 d内的死亡率不同，革兰阳性菌死亡率为10％，革兰阴性菌为22％（其中铜绿假单胞菌死亡率为67％），真菌死亡率为40％。我院血流感染阳性患者OS率较血流感染阴性患者显著减低，未培养出CRO患者OS率较培养出CRO患者高。因此在行allo-HSCT时应尽量减少血流感染的发生，减少CRO病原菌的感染。

为明确allo-HSCT患者粒缺状态对病原菌感染的影响，我们根据发生血流感染的时间对49例患者进行分组。其中在粒缺时发生血流感染的有38例（77.6％），非粒缺时发生血流感染的有11例（22.4％）。粒缺时发生血流感染的主要病原菌为革兰阴性菌，与文献[14]报道相似。非粒缺组患者OS率较粒缺组低，但差异无统计学意义。进一步对不同粒缺持续时间患者血流感染情况进行分析，结果显示粒缺持续时间>14 d的患者发生血流感染的OS率较粒缺持续时间≤14 d的患者明显减低，差异有统计学意义。结合文献和本研究结果，粒缺期患者更容易发生血流感染，但患者的转归与感染时粒缺与否相关性不大，粒缺持续时间与allo-HSCT患者血流感染及预后更为相关。

本研究对我院移植患者发生血流感染情况进行分析，但由于患者数量有限，有待后续进一步扩大样本或进行多中心研究allo-HSCT患者血流感染情况。
